# Fluconazole and Echinocandin Resistance of *Candida glabrata* Correlates Better with Antifungal Drug Exposure Rather than with *MSH2* Mutator Genotype in a French Cohort of Patients Harboring Low Rates of Resistance

**DOI:** 10.3389/fmicb.2016.02038

**Published:** 2016-12-23

**Authors:** Sarah Dellière, Kelley Healey, Maud Gits-Muselli, Bastien Carrara, Alessandro Barbaro, Nicolas Guigue, Christophe Lecefel, Sophie Touratier, Marie Desnos-Ollivier, David S. Perlin, Stéphane Bretagne, Alexandre Alanio

**Affiliations:** ^1^Laboratoire de Parasitologie-Mycologie, AP-HP, Groupe Hospitalier Saint-Louis-Lariboisière-Fernand-WidalParis, France; ^2^Public Health Research Institute, New Jersey Medical School, Rutgers Biomedical and Health Sciences, RutgersNewark, NJ, USA; ^3^Université Paris Diderot, Sorbonne Paris CitéParis, France; ^4^Service de Pharmacie, AP-HP, Groupe Hospitalier Saint-Louis-Lariboisière-Fernand-WidalParis, France; ^5^Unité de Mycologie Moléculaire, Institut Pasteur, Centre National de la Recherche Scientifique, Centre National de Référence Mycoses Invasives et Antifongiques, URA3012Paris, France

**Keywords:** *Candida glabrata*, antifungal resistance, echinocandin, fluconazole, *MSH2*, mutator genotype, genotyping, short tandem repeat

## Abstract

*Candida glabrata* is a major pathogenic yeast in humans that is known to rapidly acquire resistance to triazole and echinocandin antifungal drugs. A mutator genotype (*MSH2* polymorphism) inducing a mismatch repair defect has been recently proposed to be responsible for resistance acquisition in *C. glabrata* clinical isolates. Our objectives were to evaluate the prevalence of antifungal resistance in a large cohort of patients in Saint-Louis hospital, Paris, France, some of whom were pre-exposed to antifungal drugs, as well as to determine whether *MSH2* polymorphisms are associated with an increased rate of fluconazole or echinocandin resistance. We collected 268 isolates from 147 patients along with clinical data and previous antifungal exposure. Fluconazole and micafungin minimal inhibition concentrations (MICs) were tested, short tandem repeat genotyping was performed, and the *MSH2* gene was sequenced. According to the European Committee on Antimicrobial Susceptibility breakpoints, 15.7% of isolates were resistant to fluconazole (MIC > 32 mg/L) and 0.7% were resistant to micafungin (MIC > 0.03 mg/L). A non-synonymous mutation within *MSH2* occurred in 44% of the isolates, and 17% were fluconazole resistant. In comparison, fluconazole resistant isolates with no *MSH2* mutation represented 15% (*P* = 0.65). MSH2 polymorphisms were associated with the short tandem repeat genotype. The rate of echinocandin resistance is low and correlates with prior exposure to echinocandin. The mutator genotype was not associated with enrichment in fluconazole resistance but instead corresponded to rare and specific genotypes.

## Introduction

Ascomycetous yeasts are the most common agents responsible for fungal infections in humans, and the incidence of *Candida* spp. infections has significantly increased over the last two decades (Lass-Flörl, 2009). Up to 17% of all ICU-acquired infections are caused by *Candida* spp. and are associated with a high mortality rate, which is showing increasing trends (Lass-Flörl, 2009; Lortholary et al., 2014). *Candida glabrata* is the second most-common bloodstream *Candida* species isolated in Europe and North-America (Pfaller and Diekema, 2007). Even though *C. glabrata* cannot form hyphae, a well-known virulence factor in *C. albicans*, its pathogenic traits and rapid acquisition of resistance, especially to azoles and echinocandins, are a matter of concern (Cleveland et al., 2012; Wisplinghoff et al., 2014; Vale-Silva and Sanglard, 2015). This yeast has intrinsically low susceptibility to fluconazole, with 10–30% of C. *glabrata* isolates harboring high fluconazole MICs (MIC >32 mg/L) (Pfaller and Diekema, 2007).

The mechanisms leading to high fluconazole or echinocandin MICs have been documented (Jensen et al., 2015; Vale-Silva and Sanglard, 2015). Most published epidemiological data report the emergence of echinocandin resistance in *C. glabrata*, especially in North America. The prevalence of echinocandin resistance varies depending on the geographical location, with Europe showing lower rates (0–9%) (Klotz et al., 2016) compared to the rates of 0–25% in the USA (Vallabhaneni et al., 2015). Furthermore, echinocandin resistance seems to have increasing prevalence over time (Alexander et al., 2013; Farmakiotis et al., 2014) and to be associated with a higher mortality rate (Alexander et al., 2013; Shields et al., 2015). *C. glabrata* develops echinocandin resistance through target mutation of the 1,3-beta-D-glucan synthase gene encoded by *FKS1* and *FKS2* genes (Alexander et al., 2013; Perlin, 2015). Simultaneous resistance to all echinocandins (caspofungin, micafungin, and anidulafungin) has been detected using various susceptibility-testing methods after specific hotspot polymorphisms develop.

The reason why *C. glabrata* acquires resistance more rapidly to multiple antifungal classes than other *Candida* species is unclear and has been attributed to its haploid genome. However, Healey et al. recently suggested that a defect in DNA repair may account for accelerated emergence of various genetic changes responsible for drug resistance (Healey et al., 2016). The aim of this study is to evaluate the factors associated with the isolation of fluconazole or echinocandin resistance in a single French hospital using all isolates recovered over a specific period of time. The association between resistance and antifungal drug pre-exposure as well as *MSH2* sequence type, representing potential mutator genotypes, were carefully studied after all isolates were genotyped with short tandem repeat markers to identify individual strains of *C. glabrata*.

## Materials and methods

### Ethics statement

Saint-Louis Hospital, Paris, France, is a tertiary university hospital with major clinical activities in hematology, renal transplantation, burn unit, and oncology. This study was a non-interventional study with no change in the usual procedures. Biological material and clinical data were obtained only for standard diagnostics following physicians' prescriptions with no specific sampling. According to French Health Public Law (CSP Art L1121-1.1), such a protocol does not require the approval of an ethics committee and is exempted from specific informed consent application.

### Isolates and patients

All consecutive *C. glabrata* isolates recovered from June 2015 to February 2016 in our mycology laboratory (Saint-Louis Hospital, Paris, France) were prospectively collected. A total of 268 isolates were obtained from various anatomical sites of 147 patients (1–19 isolates per patient, Table 1). All isolates were primary cultured on CHROMagar® Candida Medium (Becton Dickinson, Heidelberg, Germany) incubated at 37°C for 5 days and identified as *C. glabrata* using matrix-assisted laser desorption/ionization time-of-flight mass spectrometry (MALDI-TOF, Vitek MS; Biomérieux, Marcy l'Etoile, France). For each isolate, a minimum of 5 colonies were picked together and stored at −80°C. DNA was extracted using a MagNA Pure LC apparatus (Roche, Mannheim, Germany) according to the manufacturer's recommendations and stored at −20°C until PCR was performed. In our hospital, each antifungal drug prescription except for fluconazole is recorded by the pharmacy department with the corresponding indication. This registry has been used to analyze the duration of previous antifungal exposure for each *C. glabrata* isolate.

**Table 1:** **Characteristics of patients and isolates**.

**Characteristics**	**Patients (*n* = 147)**	**Isolates (*n* = 268)**
Age (years), median (range)	62 (18–91)	
Sex, male/female ratio	0.91	
Sample origin, n (%)		
Abdominal (surgery)		8 (3.0)
Blood		6 (2.2)
Feces		46 (17.2)
Mouth		50 (18.7)
Respiratory		81 (30.2)
Skin		22 (8.2)
Uro-vaginal		55 (20.7)
Hospital Unit, n (%)		
Hematology	34 (23.1)	63 (23.5)
ICU	38 (25.9)	102 (38.1)
Others	75 (51)	103 (38.4)
Patients treated with voriconazole or posaconazole (Δt first day of treatment–day of sampling)		
>7 days		41 (15.3)
<7 days		11 (4.1)
Not treated		216 (80.6)
Patients treated with caspofungin (Δt first day of treatment–day of sampling)		
>7 days		23 (8.6)
<7 days		9 (3.4)
Not treated		236 (88.1)

*ICU, Intensive Care Unit*.

### Susceptibility testing

Although our hospital does not prescribe micafungin, we tested for it instead of caspofungin according to the recommendations of the Clinical and Laboratory Standards Institute (CLSI) and European Committee on Antimicrobial Susceptibility (EUCAST) (Arendrup et al., 2013). Since interlaboratory variability occurs with caspofungin testing (Espinel-Ingroff et al., 2013), micafungin or anidulafungin can serve as testing surrogates for the echinocandin class to assess resistance (Pfaller et al., 2014a,b; Perlin, 2015). In addition, high micafungin MIC results have been shown to correlate with the occurrence of *FKS* mutations, which is also the case for high caspofungin MICs (Pham et al., 2014). We used Etest® for the echinocandin susceptibility testing since it is considered a valuable alternative to the EUCAST broth microdilution method for routine susceptibility testing of micafungin in *C. glabrata* isolates, with categorical agreement of 96.7% (Bougnoux et al., 2016)

Micafungin and fluconazole MICs were prospectively determined after isolation with Etest® strips (Biomerieux, Marcy L'Etoile, France) according to the manufacturer's instructions. Briefly, a pool of five yeast colonies of each isolate was suspended in 0.9% sterile saline solution to adjust to a turbidity of a 0.5 McFarland standard (Biomérieux). *Candida krusei* ATCC 6258 and *C. parapsilosis* ATCC 22019 were used as quality controls. A broth microdilution reference was used according to the EUCAST method (Arendrup et al., 2012) to test for susceptibility to fluconazole and micafungin *in vitro* using 18 randomly chosen isolates and 2 isolates showing high micafungin MICs. AM3 medium was used for dilution of micafungin instead of RPMI due to its better ability to distinguish between mutant and wild-type isolates for fks protein (Desnos-Ollivier et al., 2008).

*C. krusei* ATCC 6258 and *C. parapsilosis* ATCC 22019 were used as quality controls. We used breakpoint tables of the EUCAST document v8.0 (http://www.eucast.org/clinical_breakpoints/), which were valid as of November 16, 2015 (European Committee on Antimicrobial Susceptibility Testing (EUCAST), 2015), and the EUCAST and Etest® database of MIC distribution and ECOFFs (http://www.mic.eucast.org). Isolates were categorized as high fluconazole MICs for MICs > 32 mg/L and high micafungin MICs for MICs > 0.03 mg/L (Espinel-Ingroff et al., 2016). Notably, some isolates, including reference strain ATCC MYA 2950 displayed macrocolonies in the inhibition ellipse of Etest® after 48 h of incubation with fluconazole. These macrocolonies were not observed with the EUCAST technique and were hence not taken into account for Etest® interpretation.

### *MSH2* sequencing

The first 156 consecutive isolates and the two isolates harboring high micafungin MICs were selected for *MSH2* sequencing. Twelve primers to cover the 2874 bp gene were used for PCR and sequencing the amplified DNA and to identify *msh2* mutations in *C. glabrata* isolates (Supplementary Table 1). Initial amplification of *MSH2* was performed using 3 couples of primers (33F/1888R; 1013F/2170R; 1914F/2985R) and sequenced with the 12 primers listed in Supplementary Table 1. PCR was performed in a volume of 50 μL using 25 μL of ampliTaq Gold 360 MasterMix (Applied Biosystems, Forster City, USA), isolate extracted DNA (1 ng/μL), forward primer (0.6 μM), and reverse primer (0.6 μM). The thermal cycling parameters were as follows: initial denaturation of 10 min at 95°C; 35 cycles of 95°C for 30 s, 55°C for 30 s, and 72°C for 1 min 30 s; and a final elongation step at 72°C for 7 min.

PCR sequencing was performed using BigDye Terminator v1.1 (Thermo) as recommended by the manufacturer. The purification of the PCR products was performed using illustra ExoProStar™ 1-Step (GE Healthcare Life Sciences, Little Chalfont, UK). Sequencing was performed with all primers described (Supplementary Table 1) and using a 3500 series sequencing analyzer (Applied Biosystems, Forster City, USA). The obtained sequences were compared with the reference wild-type sequence of *C. glabrata MSH2* (GenBank accession number XM_447585.1) using Geneious v. 8.0 software (Auckland, New Zealand). Non wild-type sequences are available under the GenBank accession numbers KY110686–KY110696.

### FKS sequencing

Twelve primers were used for sequencing the two hotspots regions of the *FKS1 and FKS2* genes as described previously (Zimbeck et al., 2010) of the two isolates harboring high micafungin MICs and 9 susceptible isolates. PCR was performed in a volume of 50 μL with 25 μL of ampliTaq Gold 360 MasterMix (Applied Biosystems, Forster City, USA), isolate extracted DNA (1 ng/μL), forward primer (0.25 μM), and reverse primer (0.25 μM). The thermal cycling parameters were as follows: initial denaturation of 10 min at 95°C; 30 cycles at 95°C for 30 s, 58°C for 30 s and 72°C for 30 s; and a final elongation step at 72°C for 10 min. The sequencing parameters where similar to those used for the *MSH2* PCR described above. The sequences were compared with those of a reference strain ATCC 2001 (*FKS1* GenBank accession number XM_446406, *FKS2* GenBank accession number XM_448401, and *FKS3* GenBank accession number HQ_845285). Newly described partial *FKS* sequences are available under the GenBank accession number KY110697.

### *MSH2* polymorphism phenotyping assay

A phenotyping assay was used to assess the impact of *MSH2* polymorphisms on the induction of echinocandin resistance as described previously (Healey et al., 2016). Isolates harboring the previously undescribed *MSH2* polymorphisms (E7K, P208S/N890I/Y949C, S591Y, E478Q, S346T, and M651T) were selected and tested. Briefly, the *MSH2* sequence was amplified from the corresponding clinical isolates, expressed on a yeast centromere plasmid under the control of their native promoter, and transformed into a laboratory strain carrying a deletion of *MSH2* according to Healey et al. (2016). Forward mutation frequencies were analyzed by measuring frequencies of colonies resistant to caspofungin in order to identify whether the tested *MSH2* polymorphism contributed to an increase in mutation rate and increased emergence of antifungal resistance.

### Short tandem repeat (STR) genotyping analysis

Ten previously described STRs markers VNTR2bis, VNTR3, VNTR4, VNTR5, VNTR6, VNTR8, VNTR9 (Brisse et al., 2009), GLM5, GLM6 (Abbes et al., 2012), and Cg6 (Grenouillet et al., 2007) were amplified. The forward primers were tagged with fluorophores (FAM, HEX, or ATTO565). All PCR reactions were performed on a GeneAmp PCR System 9700 Thermocycler (Applied Biosystems) in a final volume of 20 μL containing 1X Ampli Taq Gold buffer (Life technologies) with 0.25 μM of each primer, 2.5 mM of MgCl2, 0.8 μM of dNTPs, 0.25 UI of Ampli Taq Gold polymerase (Life technologies), and 2 μL of DNA. The reaction consisted of 10 min at 95°C; 35 cycles of 30 s at 95°C, 30 s at 55°C, and 60 s at 72°C; and a final extension at 72°C for 10 min. After amplification, 2 μL of PCR product was prepared for fragment analysis by adding 18.5 μL of formamide (3700 formamide, Life technologies) and 0.5 μL of Genescan-500 LYZ Size Standard (Life technologies). Capillary electrophoresis was performed with the denaturing polymer POP-7 (Life technologies) in a 50-mm capillary tube at 60°C. The lengths of the PCR fragments were determined on an ABI 3500 genetic analyzer with ABI Gene mapper v4.1 software (Applied Biosystems). Rare genotypes were those for which fewer than 3 isolates/patient were found in our collection. Singletons are genotypes found in only one isolate of our cohort.

### Statistical analyses and graphs

All analyses were performed with GraphPad Prism software v6.0 (GraphPad Software, San Diego, CA). Chi-square tests were used to determine differential repartition in *MSH2* mutational status, in anatomical localization in specific genotypes between susceptibility groups and to determine resistance. *P*-values were determined by a non-parametric *t*-test. A *P* < 0.05 (two-tailed) is considered statistically significant.

## Results

### Resistance rate to fluconazole and micafungin

**Figure 1:**
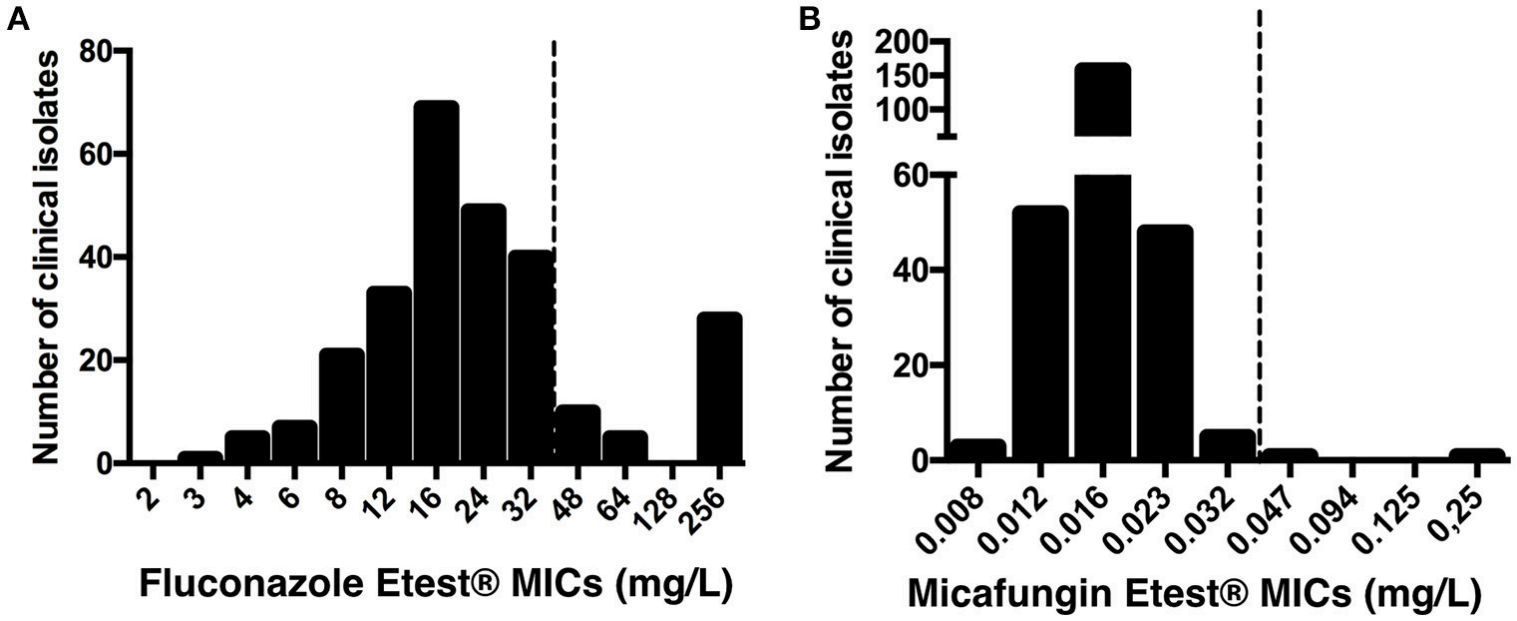
**Distribution of fluconazole (A)** and micafungin **(B)** MICs using Etest® for 268 clinical isolates of *C. glabrata* from 147 patients. Dotted line shows recently published EUCAST breakpoints for *C. glabrata* susceptibility.

Fluconazole and micafungin Etest® MICs were determined for the 268 *C. glabrata* isolates. The characteristics of the 147 patients and isolates are summarized in Table 1, and the distributions of the Etest® MICs are presented in Figure 1. MIC50 and MIC90 were 16 mg/L and 256 mg/L for fluconazole and 0.016 mg/L and 0.023 mg/L for micafungin, respectively. Fluconazole MICs > 32 mg/L occurred in 42 (15.7%) of the 268 isolates. Two isolates (0.7%) had micafungin MICs > 0.032 mg/L (isolates #96 [0.047 mg/L] and #230 [0.25 mg/L]). Isolate #96 exhibited both high fluconazole and micafungin MICs. Essential agreement (± 2 dilutions) for fluconazole and micafungin MICs was observed for the 20 isolates tested with Etest® and EUCAST methods (data not shown). EUCAST MICs for isolates #96 and #230 were 64 and 8 mg/L for fluconazole and 0.03 and 0.25 mg/L for micafungin, respectively.

The two hotspots of the three *FKS* genes of the two isolates harboring high micafungin MICs were sequenced and revealed mutation K1323T in HS2 of *FKS1* for isolate #96 and mutation FKS2-F658Δ in HS1 of *FKS2* for isolate #230. Notably, the nine randomly selected isolates without high micafungin MICs had wild-type sequence for *FKS* genes.

### Impact of drug exposure on antifungal resistance

The duration of exposure and the date of initiation of caspofungin, voriconazole, and posaconazole within the past 6 months before sampling was recorded for all patients. Pre-exposed isolates were defined as isolates recovered from patients exposed for at least 7 days to antifungal drugs (treatment began ≥ 7 days before sampling). Voriconazole and posaconazole pre-exposure was significantly associated with recovery of isolates with fluconazole MICs > 32 mg/L (*P* < 0.001). The proportion of isolates harboring fluconazole MICs > 32 mg/L was significantly higher (20/41, 48.8%) among isolates exposed to triazoles than among unexposed isolates (23/227, 10.1%). Pre-exposure to caspofungin was also significantly associated with higher occurrence of resistance to micafungin (*P* = 0.007), with two isolates having high micafungin MICs among the 23 isolates exposed to caspofungin. The patient with isolate #230 suffered from extensive burns and was treated with caspofungin and voriconazole for invasive skin infection due to molds. Recurrent mouth and skin swabs showed the emergence of echinocandin resistant *C. glabrata* after 8 days of treatment. Patient with isolate #96 received caspofungin, but data concerning duration of treatment were lacking.

### Impact of mutator genotype on antifungal resistance

*MSH2* sequencing was performed for the first 156 isolates and two micafungin resistant isolates (90 patients).Ŏverall, 69 (44%) isolates harbored non-synonymous polymorphisms within *MSH2* gene as compared with the ATCC 2001 reference strain (GenBank accession number: XM_447585.1). Non-synonymous *msh2* polymorphisms led to several amino acid changes: P208S/N890I (*n* = 17), V239L/A942T (*n* = 14), V239L (*n* = 11), E7K (*n* = 9), and E456D (*n* = 8; Table 2). One isolate harboring a pure microsatellite genotype (one allele per marker) had two different *MSH2* genotypes P208S/N890I and V239L/A942T upon sequencing (double peaks at specific positions). Notably, micafungin-resistant isolates #96 and #230 displayed P208S/N890I and wild-type *MSH2* sequences, respectively.

**Table 2:** **Fluconazole MIC distribution of 158 isolates according to *MSH2* genotype**.

**Etest MICs (mg/L)**	**WT**	**P208S/N890I**	**V239L/A942T**	**V239L**	**E7K**	**E456D**	**M651T**	**S591Y**	**V34E/V239L/A942T**	**P208S/N890I/Y949C**	**S346T**	**E478Q**
GenBank accession number		KY110692	KY110686	KY110688	KY110687	KY110690	KY110696	KY110689	KY110693	KY110691	KY110694	KY110695
%R	14.8	23.5	0.0	18.2	11.1	0.0	100.0	0.0	100.0	0.0	0.0	100.0
3			1									
4	5											
6	3		2	2								
8	8	1	5									
12	9	3	1	2	3	1		1				
16	17	3^*^	3^*^	3	3	2				1	1	
24	16	2	2	2	1	4		1				
32	18	5	1		1	1						
48	3	2		1								
64	4											
256	6	2		1	1		3		1			1
Total	89	18^*^	15^*^	11	9	8	3	2	1	1	1	1

*WT, wild-type; %R, percentage of isolates resistant to fluconazole (CMI > 32 mg/L) among genotype*.

* *Of note, one isolate (Fluconazole MIC = 16mg/L) harbored a mixture of the P208S/N890I and V239L/A942T MSH2 sequences and a unique microsatellite genotype (Gt18)*.

Overall, *MSH2* non-synonymous polymorphisms were observed in 48% (12/25) of the isolates harboring high fluconazole MICs and 42.8% (57/133) of isolates harboring low fluconazole MICs (*P* = 0.66). To avoid any biases in this analysis, we selected one more isolate per genotype per patient (111 isolates, 90 patients) in order to avoid counting the same genotype several times in a given patient. In this situation, no significant association was observed (*MSH2* non-synonymous polymorphisms were observed in 62.5% (10/16) of the isolates harboring high fluconazole MICs and 49.5% (47/95) of isolates harboring low fluconazole MICs (*P* = 0.42)). To enable comparison with data presented in Healey et al. (2016), no significant association was also observed when the threshold for fluconazole resistance was considered as MIC ≥ 32 mg/L.

### Relationship between mutator genotype and short tandem repeat genotype

Genotyping analysis of the 268 isolates determined by validated microsatellite genotyping (10 markers) revealed the presence of 49 genotypes (Gt) (median number of isolates per genotypes = 1, range = 1–18). Rare (≤3 isolates per genotype) and frequent (>3 isolates per genotype) genotypes represented 43 minor genotypes (median 1, interquartile [1–1]) and 9 major genotypes (median 4, interquartile [3–6], range [1–18]). Among the 156 isolates sequenced for *MSH2*, 101 isolates, corresponding to one isolate per genotype per patient, were selected to analyze the correlation between microsatellite genotype and *MSH2* sequence. From these 49 genotypes, 44 were considered rare (≤3 isolates per genotype) and 5 frequent (>3 isolates per genotype). Association between *MSH2* polymorphism and genotype exists since in the 17 genotypes found more than once in our collection, 13 (76%) harbored the same *MSH2* polymorphism in all isolates from a specific genotype (Table 3).

**Table 3:** **Genotype distribution of 101 isolates (one isolate per genotype per patient) according to the most frequent *MSH2* genotype**.

	**Gt (n)**	**WT n (%)**	**E7K n (%)**	**P208S/N890I n (%)**	**E456D n (%)**	**V239L/A942T n (%)**	**V239L n (%)**	**Other n (%)**
Frequent genotypes (5 genotypes, 41 isolates)	Gt1 (19)	15 (88)	3 (16)					1 (6) S346T
	Gt2 (4)	4 (100)						
	Gt10 (5)					4 (80)		1 (10) V34E/V239L/A942T
	Gt18 (5)			5 (100)				1 (10) N890I/A942T^*^
	Gt30 (8)				8 (100)			
Rare genotypes (44 genotypes, 60 isolates)	Gt4 (3)						3 (100)	
	Gt32 (3)			3 (100)				
	Gt36 (3)	2 (66.6)		1 (33.3)				
	Gt45 (3)	3 (100)						
	Gt3 (2)	2 (100)						
	Gt5 (2)						2 (100)	
	Gt6 (2)						2 (100)	
	Gt7 (2)					2 (100)		
	Gt8 (2)					2 (100)		
	Gt22 (2)	1 (50)		1(50)				
	Gt23 (2)	2 (100)						
	Gt41 (2)						2 (100)	
	Singletons (32)	19 (59)		7 (22)		2 (6)		1 (3) P208S/N890I/Y949C; 2 (6) M651T; 1 (3) E478Q

* *One isolates harboring Gt18 was a mixture of N890I/A942T and P208S/N890I sequences*.

In our collection, wild-type isolates harbored 26 different genotypes (including 19 singletons). We found the E7K isolates in one genotype (Gt1), P208/N890I in 11 genotypes (including 7 singletons), E456D in 1 genotype (Gt30), V239L/A942T in 6 genotypes (including 2 singletons), and V239L in 4 genotypes. The seven isolates harboring other *MSH2* sequences were found in 7 genotypes (including 4 singletons). Notably, M651T isolates were found in 2 singletons. In total, there was a significant differential distribution of *MSH2* polymorphisms in rare and frequent genotypes (*P* = 0.004, Table 3).

### Impact of *MSH2* mutations on mutator phenotype

**Figure 2:**
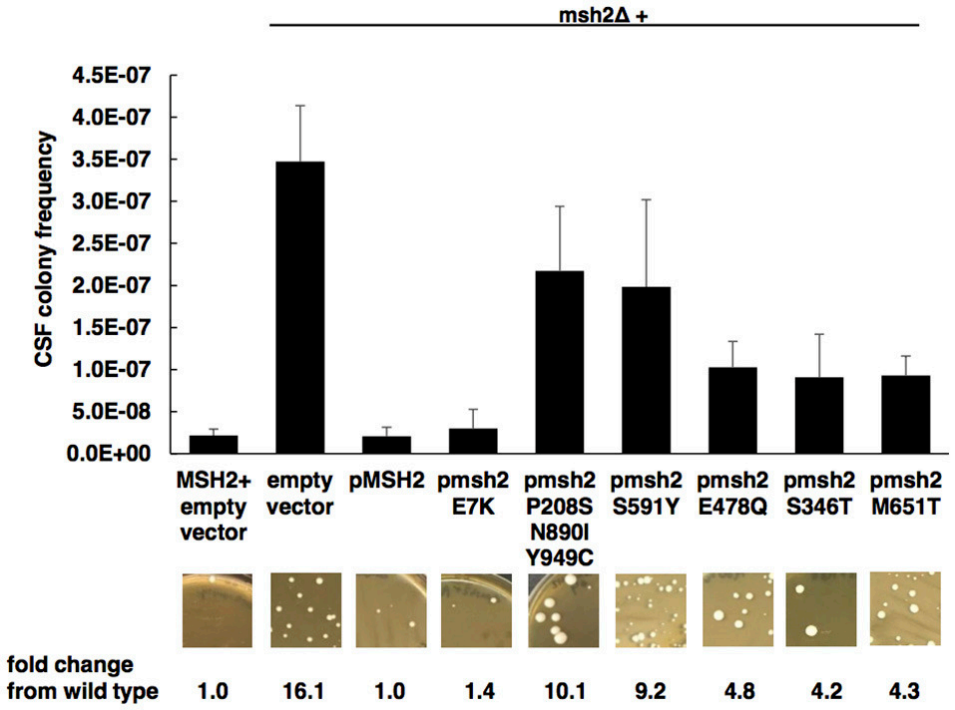
**Impact of *MSH2* mutations on mutator phenotype**. Wild-type or *MSH2*Δ cells expressing an empty or *MSH2*-containing plasmid were selected on caspofungin. Frequency data are mean ± standard deviation (s.d.) from three independent experiments; representative images are shown. *P*-values determined by student's *t*-test. A *P* < 0.05 (two-tailed) is considered statistically significant.

To assess the impact of *MSH2* polymorphisms on the induction of echinocandin resistance, a phenotyping assay was used as previously described (Healey et al., 2016). Wild-type or *MSH2*Δ cells expressing an empty or mutated *MSH2*-containing plasmid were selected on agar plates containing 1 mg/L of caspofungin (8 to 32-fold above wild type MICs). Upon caspofungin selection *in vitro, MSH2*Δ exhibited a hyper-mutable phenotype consistent with previous findings (Healey et al., 2016) (average caspofungin resistant colony frequency = 3.47E-7 ± 6.64E-8). Several *MSH2* polymorphisms (E478Q, S346T, M651T) found in our clinical isolates demonstrated moderate increases in caspofungin-resistant colony frequencies (4- to 5-fold vs. wild type), although the differences were not significant (*P* > 0.05) (Figure 2, Supplementary Table 2). However, the *MSH2*-P208S/N890I/Y949C and *MSH2*-S591Y alleles did exhibit greater increases (9 to 10-fold vs. wild type) in colony frequencies and an increasing trend (average caspofungin resistant colony frequency = 2.17E-7 ± 7.65E-8 and 1.98E10-7 ± 1.04E-7; *P* = 0.07 and *P* = 0.06, respectively). *MSH2*-E7K yielded caspofungin resistant colonies similar to wild type.

## Discussion

In our cohort, we found an intermediate level of resistant isolates to fluconazole (15.7%) and low level of resistance to micafungin (0.7%) using Etest® testing and EUCAST breakpoints, with only 1/268 isolates showing cross-resistance to both antifungal classes. There were 28 patients (19.0%) with at least one isolate with high fluconazole MIC, one patient (0.7%) with high micafungin MIC, and one patient (0.7%) with high fluconazole and high micafungin MICs. The resistance rate of *C. glabrata* to fluconazole (15.7%) builds on previous studies showing similar rates. Worldwide studies from 1997 to 2005 describe resistance rates of 14.3–19.2% with no consistent trend toward increasing resistance to fluconazole (Pfaller et al., 2007).

The prevalence of echinocandin resistance is consistent with that found in other European studies. Studies showed 1.5% resistance in a cohort of 64 bloodstream isolates in two university hospitals in Germany and Austria (Klotz et al., 2016), none in Spain and Italy from 79 bloodstream isolates (Bassetti et al., 2013), 2.1% of 94 invasive isolates obtained from Lombardy, Italy (Tortorano et al., 2013), and 1% of 193 isolates obtain from urine samples from France (Bourgeois et al., 2014). In the USA, the prevalence was between 2 and 4% to echinocandins from 1997 to 2000 (Castanheira et al., 2016).

The alarming increase in echinocandin resistance observed in the USA began in the last 10 years (Alexander et al., 2013; Vallabhaneni et al., 2015), but it does not seem to concern Europe. In addition, the prevalence in the USA seems to change from one center to another with a proportion varying from 0 to 25% of resistant isolates (Vallabhaneni et al., 2015), depending on the patient population and the presence of specific antifungal stewardship (Pfaller and Diekema, 2007; Vallabhaneni et al., 2015). The main risk factor for developing resistance to echinocandin is prior and prolonged exposure (Thompson et al., 2008; Dannaoui et al., 2012; Beyda et al., 2014; Vallabhaneni et al., 2015). This is well illustrated in our cohort, with two patients showing echinocandin resistant isolates out of 23 pre-exposed to echinocandin. However, it mostly required prolonged and repeated treatment (Thompson et al., 2008; Fekkar et al., 2014) with echinocandins, it can emerge shortly after treatment initiation (Lewis et al., 2013).

*FKS*-sequencing of isolate #230 revealed a previously described mutation in *FKS2* (Fks2-658delF) (Jensen et al., 2015; Healey et al., 2016). The *FKS1* K1323T mutation found in #96 has never been described to our knowledge. This mutation is located upstream of the DWVRRYTL *FKS1* hotspot, suggesting an implication in echinocandin resistance, but a glucan synthase inhibition assay should be implemented to help confirm the resistance.

We observed an increased proportion of colonizing isolates with high fluconazole MICs in patients who received voriconazole or posaconazole for more than 7 days. Indeed, all patients at risk of candidiasis under triazole prophylaxis or treatment for invasive or non-invasive fungal infections should be considered at risk for fluconazole resistant *C. glabrata* isolates. The kinetics of disappearance of these resistant colonizing isolates, whether they are resistant to azoles or echinocandins, is not known and would not be predictable. An important bias of our study is the lack of data concerning the use of fluconazole, the most common azole antifungal agent, because the consumption of this molecule is not prospectively monitored in our hospital. This should underestimate the rate of resistance acquired in isolates pre-exposed to triazoles.

The association of *MSH2* mutation and mutator phenotype with an increased emergence of antifungal resistance has recently been established (Healey et al., 2016). However, this study did not focus on clinical facts and treatment history, although individual center differences were noted. Several centers did not demonstrate significant differences between resistant and susceptible isolates when grouped according to *MSH2* genotype, while others did (Healey et al., 2016). Indeed, *MSH2* mutation might be better associated with multidrug resistance, which was not observed in this study. Among the most frequent mutations, P208S/N890I, V239L, and E231G/L269F were associated with an increased emergence of caspofungin-resistant colonies, while E456D was not (Healey et al., 2016). They observed a greater rate of *MSH2* mutation among fluconazole-resistant (≥32 mg/L) isolates than among susceptible ones (65 vs. 52%; *P* = 0.04).

In our study, using cut-offs of >32 mg/L (EUCAST recommendation) and ≥32 mg/L (Healey et al., 2016), we did not find a significant association between *MSH2* mutations and fluconazole resistance, although we observed the same most frequent mutations in our isolates, P208S/N890I, V239L/A942T, and V239L. However, we did not observe the mutation E231G/L269F and found one unknown mutation (E7K) in notable proportion that had not been previously analyzed for its role in MMR activity. E7K isolates were associated with a rate of 11% fluconazole-resistant strains (Table 3). Experimentally, E7K mutator phenotype is similar to that of the wild-type and E456D isolates, suggesting a conservative mutation (Healey et al., 2016).

Although the P208S/N890I/Y949C and S591Y mutants show a slight increase in frequency of caspofungin-resistant colonies, they could not be assigned to the mutator phenotype. Other mechanisms could rely on other genes involved in mismatch repair, such as PMS1 or RAD50, a double strand break repair gene. Both genes were implicated in the mutator phenotype in *C. albicans* (Legrand et al., 2007). Considering the small proportion (2/268) of isolates showing resistance to echinocandin out of our 268 isolates, we were not able to interpret the impact of *MSH2* mutations on echinocandin resistance. Microsatellite genotyping analysis of the isolates showed a significant association between *MSH2* mutations and the short tandem repeat genotypes (frequent or rare) (*P* = 0.009, Table 3). This suggests that *MSH2* could be a genotyping hallmark rather than an indicator of fluconazole resistance acquisition risk. Indeed, *MSH2* could be considered as one of the yeast's housekeeping genes, and sequence variations in this gene could be related to differences in the genetic complex rather than a direct link to fluconazole resistance.

Alternatively, the elevated rates of *MSH2* genotypes that do demonstrate partial loss of function may facilitate the pathogenesis of *C. glabrata* through acquisition of mutations that increase fitness or virulence. In agreement with the data presented here, Healey and colleagues recently described that *MSH2* genotypes largely correspond to strain type as measured by multi-locus sequence typing (Healey et al., unpublished data). Additionally, sequence types associated with *MSH2* partial loss of function genotypes, including E231G/L269F (ST16) and P208S/N890I (ST10), are among the most frequently identified in at least two American cities (Lott et al., 2010). This further highlights the differences in strain distribution between the U.S. and Europe and potentially provides insight into differences in resistance rates.

In summary, we found a low prevalence rate of resistance to echinocandins and fluconazole for *C. glabrata* in comparison with data from North America. In our study, resistance was a consequence of previous exposure to antifungal drugs. This difference in prevalence should be explained by different medical care habits and recommendations. *MSH2* mutations alone cannot explain why *C. glabrata* exhibits a rapid acquisition of resistance compared to other *Candida* spp. *MSH2* seems to be associated with the STR genotype and could be a simple marker of the genetic group of a strain.

## Author contributions

SD, KH, SB, DP and AA conceived and designed the experiments. SD, KH, BC, AB, and MG performed the experiments. SD, MG, CL, ST, NG, MD and AA analyzed the data. SD and AA wrote the manuscript. KH, SB, MD, and DP reviewed the manuscript. All authors read and approved the final manuscript.

### Conflict of interest statement

The authors declare that the research was conducted in the absence of any commercial or financial relationships that could be construed as a potential conflict of interest.
