# Supplementary material for: Fluconazole and Echinocandin Resistance of Candida glabrata Correlates Better with Antifungal Drug Exposure Rather than with MSH2 Mutator Genotype in a French Cohort of Patients Harboring Low Rates of Resistance
Source: Front Microbiol. 2016 Dec 23;7:2038. doi: 10.3389/fmicb.2016.02038 (PMC5179511; doi:10.3389/fmicb.2016.02038)
Supplement: Supplementary file 1 [file Table1.docx]

Supplementary Table 1. *MSH2* and FKS primers

| **Name** |  | **DNA sequence (5’ ->3’)** |
| --- | --- | --- |
| 33F | MSH2 | ACGTTTGTGCTATTTATTGTTGG |
| 661R |  | AGACATTCTCTTATTCCTAACTGT |
| 543F |  | TTGGGGTTGACATTCATTGA |
| 1188R |  | CGAGATTGTGACGAACTTGA |
| 1013F |  | CACTTTCTTTACGCCAATGC |
| 1631R |  | TCCTAAATCTTCTGCAGCTTC |
| 1530F |  | CGTGTGGAGTTTAATGAAGAAC |
| 2170R |  | CCCACCTGCCTGATATATGT |
| 1914F |  | ATTGCGCACTTGGATGTATT |
| 2455R |  | GCAAAACATCTAATTTCTTTTGC |
| 2354F |  | CATTATTGTTGATGAGCTTGGA |
| 2985R |  | ACATTCCCGTTATCAACAGT |
| FKS1HS1F (1) | FKS | CCATTGGGTGGTCTGTTCACG |
| FKS1HS1R (1) |  | GATTGGGCAAAGAAAGAAATACGAC |
| FKS1HS2F (1) |  | GGTATTTCAAAGGCTCAAAAGGG |
| FKS1HS2R (1) |  | ATGGAGAGAACAGCAGGGCG |
| FKS2HS1F (1) |  | GCTTCTCAGACTTTCACCG |
| FKS2HS1R (1) |  | CAGAATAGTGTGGAGTCAAGACG |
| FKS2HS2F (1) |  | TCTTGACTTTCTACTATGCG |
| FKS2HS2R (1) |  | CTTGCCAATGTGCCACTG |

(1) (Zimbeck et al., 2010)

Supplementary Table 2. Role of *msh2* mutations in mutator phenotype.

|  |  | *Msh2Δ* + | | | | | | | |
| --- | --- | --- | --- | --- | --- | --- | --- | --- | --- |
|  | *MSH2* +  empty vector | Empty  vector | p*MSH2* | p*msh2*  E7K | p*msh2*  P208S  N890I  Y949C | p*msh2*  S591Y | p*msh2*  E478Q | p*msh2*  S346T | p*msh2*  M651T |
| Average caspofungin 1 mg/L  Colony frequency | 2.16E^-08^ | 3.47E^-07^ | 2.07E^-08^ | 3.00E^-08^ | 2.17E^-07^ | 1.98E^-07^ | 1.03E^-07^ | 9.09E^-08^ | 9.29E^-08^ |
| Standard deviations | 7.78E^-09^ | 6.64E^-08^ | 1.08E^-08^ | 2.26E^-08^ | 7.65E^-08^ | 1.04E^-07^ | 3.05E^-08^ | 5.13E^-08^ | 2.31E^-08^ |
| Fold change from WT | 1.0 | 16.1 | 1.0 | 1.4 | 10.1 | 9.2 | 4.8 | 4.2 | 4.3 |

WT: wild type
